# Incidence of Tick-Borne Encephalitis during the COVID-19 Pandemic in Selected European Countries

**DOI:** 10.3390/jcm11030803

**Published:** 2022-02-02

**Authors:** Zbigniew Zając, Katarzyna Bartosik, Joanna Kulisz, Aneta Woźniak

**Affiliations:** Department of Biology and Parasitology, Faculty of Health Sciences, Medical University of Lublin, Radziwiłłowska 11 Street, 20-080 Lublin, Poland; katarzyna.bartosik@umlub.pl (K.B.); joanna.kulisz@umlub.pl (J.K.); aneta.wozniak@umlub.pl (A.W.)

**Keywords:** tick-borne encephalitis, tick-borne diseases, ticks, *Ixodes ricinus*

## Abstract

*Ixodes ricinus* ticks are one of the most important vectors and reservoirs of infectious diseases in Europe, and tick-borne encephalitis (TBE) is one of the most dangerous human diseases transmitted by these vectors. The aim of the present study was to investigate the TBE incidence in some European countries during the COVID-19 pandemic. To this end, we analyzed the data published by the European Center for Disease Prevention and Control (ECDC) and Eurostat on the number of reported TBE and COVID-19 cases in 2020 and TBE cases in 2015–2019 (reference period). Significant differences in the TBE incidence were found between the analyzed countries. The highest TBE incidence was found in Lithuania (25.45/100,000 inhabitants). A high TBE incidence was also observed in Central European countries. In 12 of the 23 analyzed countries, there was significant increase in TBE incidence during the COVID-19 pandemic during 2020 compared to 2015–2019. There was no correlation between the incidence of COVID-19 and TBE and between the availability of medical personnel and TBE incidence in the studied countries. In conclusion, Central Europe and the Baltic countries are areas with a high risk of TBE infection. Despite the COVID-19 pandemic and imposed restrictions, the incidence of TBE is increasing in more than half of the analyzed countries.

## 1. Introduction

Ticks (Acari: Ixodida) are one of the most important vectors and reservoirs of infectious and parasitic diseases in Europe [1]. *Ixodes ricinus* ticks, i.e., representatives of a group of more than 40 species of arthropods in the ixodid tick fauna occurring on the European continent, have great medical and epidemiological importance [2,3]. This is mainly related to the host preferences [4,5] and wide occurrence range of this species [2,6], as well as the spectrum of transmitted pathogens, e.g., *Borrelia burgdorferi* s.l. spirochetes causing Lyme borreliosis (LB); tick-borne encephalitis virus (TBEV), i.e., a causative agent of tick-borne encephalitis (TBE); and other less frequent tick-borne pathogens infecting humans, e.g., *Rickettsia* spp. (tick-borne Rickettsial Spotted Fever Group; RSFG), *Anaplasma phagocytophilum* (human granulocytic anaplasmosis; HGA), or *Francisiella tularensis* (tularemia), where infection via a tick bite is only one of the possible transmission routes [1,7,8,9,10]. An important role in the circulation and maintenance of tick-borne pathogens in the environment that are potentially dangerous to human health is played by other tick species occurring in Europe [1]. *Dermacentor reticulatus* ticks are capable of transmitting TBEV [11]. In turn, the presence of the genetic material of this virus in *D. marginatus* has been confirmed, but the role of this species as a competent TBEV vector has not been elucidated [12].

Next to LB, TBE is one of the most commonly diagnosed tick-borne diseases in Europe [13,14]. In recent years, a systematic increase in the incidence of this disease has been reported in many countries. Austria, the Czech Republic, Germany, Lithuania, Latvia, Estonia, southern Scandinavia, northeastern Poland, and the European part of Russia, where *I. persulcatus* is another TBEV vector, should be considered as high TBE risk areas [15,16,17].

Based on molecular studies, three main genetic types of TBEV have been distinguished, including European, Siberian and Far Eastern types. Additionally, detailed studies showed the existence of other subtypes, e.g., 886 strand group, reported form southern Siberia [18]. TBEV Far Eastern type, common in eastern Russia, South Korea, and China is associated with complications in 60% of patients and mortality up to 20%, whereas the Siberian type reported from central and western Russia and Eastern Europe mortality rate is estimated at 6–8%. Therefore, the European TBEV subtype seems to be less virulent, with a mortality rate of 1–2% and symptoms developing in 1/3 of infected subjects [19,20,21].

The European type of TBE is most often characterized by a biphasic course. The viremic period, which lasts for several days, is associated with flu-like symptoms followed by a period of apparent improvement. The second phase develops after the entry of the virus into the central nervous system in approximately 30% of patients. It is manifested by recurrent high fever, headaches, dizziness, and vomiting. Depending on the infected area in the nervous system, further symptoms may appear. Patients with meningitis (the most common form of TBE) develop photosensitivity and neck stiffness without consciousness impairment, which is characteristic of the less common encephalomyelitis and/or spinal cord inflammation. Additionally, patients present with ataxia, agitation, hyperkinesia of limb and facial muscles, convulsions, and speech disorders; mental disorders are also possible. The elderly are especially vulnerable to the severe course of TBE. Children often develop asymptomatic infections. Post-inflammatory complications develop in up to 60% of patients [14,20,22,23,24]. The TBE prophylaxis methods include the use of repellents and avoidance of tick habitats. The most effective method is vaccination, which provides protection at the level of 98% after the full cycle [20,25,26].

Both, TBE and COVID-19 are seasonal viral diseases. In the case of TBE, cyclic variations are determined by the rhythms of seasonal activity, abundance and population size of the main vector (depending on the region *I. ricinus* or *I. persulcatus* or both), whereas in the case of COVID-19, the current course of the pandemic suggests that periodic increases in the incidence of this disease in Europe are observed in the autumn–winter period, i.e., similar to other seasonal viral diseases affecting the respiratory system [27,28,29].

The global COVID-19 pandemic, which developed towards the end of 2019 in China, and in Europe in March 2020, has resulted in limitations in the access to primary and specialized medical care in many countries [30,31]. This may increase the incidence of various diseases in the future (due to late diagnosis), including tick-borne diseases [32,33]. The aim of the present study was to investigate the TBE incidence in some European countries during the COVID-19 pandemic in 2020.

## 2. Materials and Methods

The study covered the countries of the European Union and the European Economic Area, which in 2015–2020 reported on the number of infected patients and TBE incidence to the European Center for Disease Prevention and Control (ECDC). In total, 23 countries were analyzed: Austria, Belgium, Bulgaria, Croatia, Czech Republic, Estonia, Finland, France, Germany, Greece, Hungary, Ireland, Italy, Latvia, Lithuania, Luxembourg, Norway, Poland, Romania, Slovakia, Slovenia, Spain, and Sweden.

### 2.1. COVID-19 Incidence

Data on the number of COVID-19 cases in 2020 and the number of inhabitants in the studied countries were obtained from the statistical office of the European Union (Eurostat) [34,35]. The data were used to calculate the incidence of COVID-19 per 100,000 inhabitants in each country according to the formula:

$$I=\frac{k}{p}\times 100,000$$

where:

*I*—incidence per 100,000 inhabitants;
*k*—number of recorded cases,
*p*—total number of inhabitants.

### 2.2. TBE Incidence

The data on the number of TBE cases were obtained from ECDC [36]. Two periods were analyzed: the pre-pandemic period (2015–2019) and the COVID-19 pandemic year (2020). The incidence rate per 100,000 inhabitants was calculated as above (Section 2.1). Additionally, based on the data from 2015–2019, the projected TBE incidence in 2020 was calculated.

Moreover, Eurostat data on the number of medical personnel in the countries were analyzed [37].

### 2.3. Statistical Analysis

The measurable variables of TBE incidence in the analyzed countries were described using the median due to their right-skewed distribution.

The distribution of the data was verified using the Shapiro–Wilk test. The statistical significance of differences in the TBE incidence between the studied countries over the years was verified by the Friedman ANOVA test with the post hoc Dunn test. The differences between the projected and reported TBE incidence rates were tested using the pairwise Wilcoxon test. The Spearman rank correlation coefficient was used to test the relationships between the number of healthcare staff per 100,000 inhabitants in the analyzed countries and the TBE incidence, and between the incidence of COVID-19 and TBE. The significance of the differences in the TBE incidence between 2015–2019 and the pandemic year (2020) was verified by a one-sample t-test.

The value of *p* < 0.05 was considered statistically significant. Statistical calculations were performed using the STATISTICA 13 PL statistical package (StatSoft, TIBCO Software Inc., Palo Alto, CA, USA).

## 3. Results

### 3.1. TBE Incidence in 2015–2019

**Figure 1:**
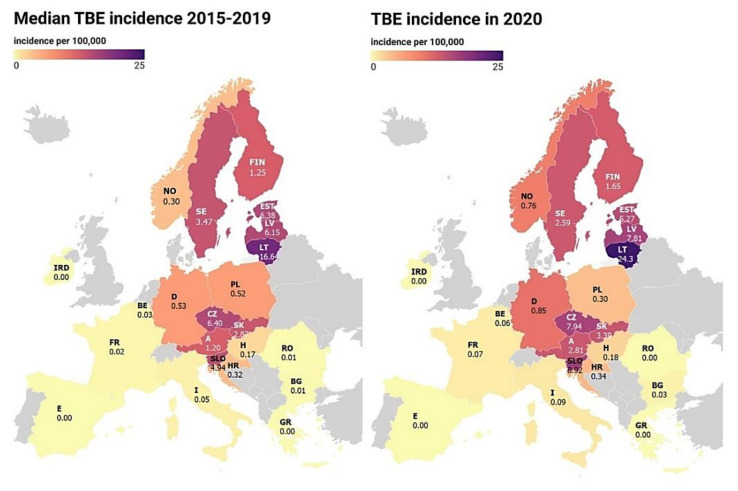
Median TBE incidence in some European countries in 2015–2019 and TBE incidence in the pandemic year 2020. Abbreviations: E—Spain, FR—France, I—Italy, IRD—Ireland, BE—Belgium, D—Germany, A—Austria, SLO—Slovenia, HR—Croatia, H—Hungary, RO—Romania, BG—Bulgaria, GR—Greece, SK—Slovakia, CZ—Czech Republic, PL—Poland, LT—Lithuania, LV—Latvia, EST—Estonia, FIN—Finland, SE—Sweden, NO—Norway.

In 2015–2019, the TBE incidence in the analyzed countries ranged from 0.00 to 25.45/100,000 inhabitants and were statistically, significantly different between the countries (χ^2^ = 13.25, *p* = 0.0101). A growing trend was noted between the subsequent years (Table 1). The highest incidence was recorded in the Baltic republics and Central European countries. The highest five-year incidence median was reported in Lithuania (16.64/100,000 inhabitants) and in the Czech Republic (6.40/100,000 inhabitants). The median incidence exceeding 5.00/100,000 inhabitants was also noted in Estonia and Latvia. The lowest incidence rates were recorded in Western Europe. The median incidence was 0.02/100,000 inhabitants in France and 0.05/100,000 inhabitants in Italy. In the group of countries reporting tick-borne diseases to ECDC, no TBE infection was confirmed in Greece, Spain, and Ireland in 2015–2019 (Figure 1).

### 3.2. TBE Incidence in the Pandemic Year 2020

**Figure 2:**
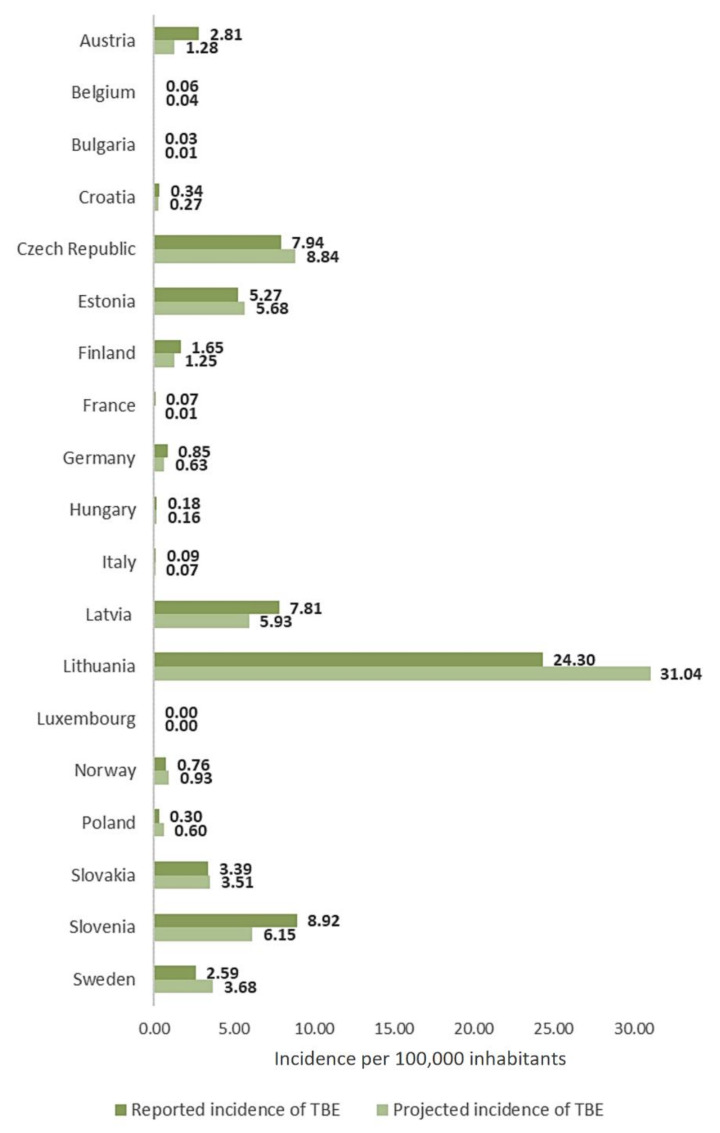
Projected and reported TBE incidence in the analyzed countries. The graph presents countries reporting TBE cases.

The projected TBE incidence in 2020 in the studied countries ranged from 0.00/100,000 inhabitants in Luxembourg to 31.04/100,000 inhabitants in Lithuania. In turn, the reported incidence reached 24.3/100,000 inhabitants in Lithuania. There was no significant difference between the predicted and reported values (Z = 0.15, *p* = 0.8789) (Figure 2).

**Figure 3:**
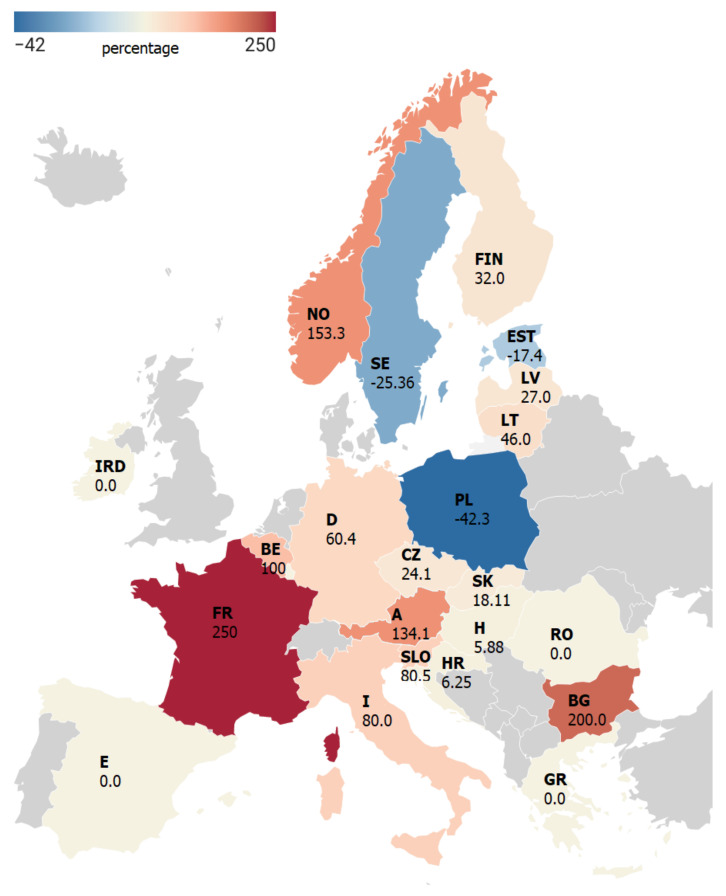
Percentage change in the TBE incidence per 100,000 inhabitants in 2020 compared to the 2015–2019 median in the analyzed countries. Abbreviations: E—Spain, FR—France, I—Italy, IRD—Ireland, BE—Belgium, D—Germany, A—Austria, SLO—Slovenia, HR—Croatia, H—Hungary, RO—Romania, BG—Bulgaria, GR- Greece, SK—Slovakia, CZ—Czech Republic, PL—Poland, LT—Lithuania, LV—Latvia, EST—Estonia, FIN—Finland, SE—Sweden, NO—Norway.

As in 2015–2019, the analyzed countries were characterized by a similar distribution of the incidence values (Table 1, Figure 1). In the case of Austria, Belgium, Bulgaria, Finland, France, Germany, Italy, Norway, and Slovenia, there was a statistically significant increase in the TBE incidence in 2020 versus the incidence in 2015–2019 (Table 2). In comparison with the reference period, the increase in the TBE incidence in these countries ranged from 32.0% (Finland) to 250.0% (France) (Figure 3). In turn, a significantly lower incidence rate in 2020 than in the reference period 2015–2019 was observed in Poland (−42.3%) and Estonia (−17.4%) (Figure 3, Table 2). Nevertheless, in all the countries, there was no significant difference in the TBE incidence between 2020 and 2015–2019 (Z = 1.93, *p* = 0.0534).

In the analyzed countries, there was no correlation between the availability of medical personnel and TBE incidence (r_s_ = 0.151, *p* = 0.6403), or between the incidence of COVID-19 regarded as a factor reducing access to healthcare and TBE incidence (r_s_ = 0.129, *p* = 0.5572).

## 4. Discussion

Tick-borne diseases are one of the most significant infectious diseases in Europe [13,38]. The results of the present study show that TBE is an especially important and growing problem in the Baltic, Central European, and Scandinavian countries, as the incidence of this disease in the analyzed period reached as high as 24.3/100.00 inhabitants in Lithuania (Figure 1). In many European countries, TBE is the second-most common human tick-borne disease after LB in terms of the number of diagnosed cases [39,40,41,42]. Microclimatic conditions are the main factors that support the activity of ticks and, in an indirect way, the transmission of tick-borne pathogens [43]. Nevertheless, an important role in formation and stability of TBE natural foci plays coincidence of several ecological factors such as air temperature and air relative humidity, soil humidity, vegetation, type of the biotope, population density and the dynamics of seasonal activity of ticks and their hosts. Significant factors influencing TBEV circulation in the zoonotic cycle are hosts’ immunological statuses, their susceptibility to TBEV infection and the prevalence of this virus in reservoir animals [44,45,46,47,48]. The shorter winter period accelerates and extends the period of tick activity, thus contributing to a higher survival rate of ticks [49] and their potential hosts [50]. Such conditions have recently been observed in Central Europe [51], i.e., the region with the highest TBE incidence on the continent. The significant differences in the incidence of TBE between the analyzed European countries shown in the present study may primarily be caused by the location of these countries in different climatic zones. The lowest TBE incidence is reported in Southern European countries with a dry climate (Figure 1, Table 1). Such conditions may exert an adverse effect on the number of potential hosts and reduce the level of infestation with juvenile tick stages [49]. With sufficient reserve materials, *I. ricinus* larvae and nymphs, which are sensitive to water loss, avoid unfavorable conditions. This shortens the host questing time and reduces the probability of TBEV transmission in the rodent (reservoir)–tick (vector) system [49,52]. Another possible route of TBEV infection is the consumption of unpasteurized dairy products [53]. Nevertheless, in our opinion, based on current sanitary restrictions on the sale of unpasteurized milk, this route of infection accounts for a negligible percentage of all TBE cases.

The countries analyzed in the present study were characterized by an uneven distribution of TBE incidence between the sub-regions [15]. This phenomenon was particularly evident in the case of Sweden, Norway (most TBE cases were reported from southern regions), Germany (Bavaria), and Poland, where the northeastern part of the country is the endemic TBE region. These regions are characterized by a large proportion of forest cover [54,55,56]. Our earlier study conducted in eastern Poland showed a significant positive correlation between TBE and LB prevalence rates and the surface area of forests [42]. Regional differences in TBE prevalence have also been reported in the Czech Republic, Austria, and Slovakia [57,58,59].

The present results indicate the highest TBE incidence in the Baltic republics, both in the reference period and in the pandemic year (2020; Table 1, Figure 1). This situation has been noted for many years. During the period 1990–2000, Latvia reported the highest TBE incidence in the world (up to 53/100,000 inhabitants) [60]. Very high TBE incidence (up to 40.3/100,000 inhabitants) and death rates were also reported in Lithuania [61,62]. The very high TBE incidence in these countries is most probably associated with specific microhabitat conditions and the presence of *I. persulcatus* ticks [63,64]. In this region, approx. 26.6% of *I. ricinus* ticks and 37.3% of *I. persulcatus* ticks collected from the vegetation were infected with TBEV. In turn, TBEV was detected in nearly 30% of ticks removed from humans [64]. Notably, these countries have the highest TBE vaccination rates, i.e., 53% of the population in Latvia [64], compared to 3% in Germany [65] and approx. 10% in Slovakia (based on self-report surveys) [66].

The results of the present study show that the reported TBE incidence in the analyzed countries in 2020 did not differ significantly from the projected incidence values (Figure 2). Moreover, the TBE incidence increased in more than half of the analyzed countries in 2020 (of which a statistically significant increase was observed in 12 countries (Table 2)), compared to the reference period, with the largest increase in France and Bulgaria (250% and 200%, respectively) (Table 2, Figure 3). The very high increase in the TBE incidence in these countries is, however, related to the very low baseline value in the reference period (Table 2). Nevertheless, the increase in the number of TBE cases was noted across the continent despite the COVID-19 pandemic and government-imposed lockdowns.

A decrease in TBE incidence in 2020 compared to the reference period was recorded in only three countries, i.e., Poland (−42.3%), Sweden (−25.3%), and Estonia (−17.4%) (Figure 3). The decrease was statistically significant in the case of Poland and Estonia (Table 2). We believe that this may be associated with the change in the seasonal dynamics of ticks and/or the restrictions on movement. For instance, the so-called hard lockdown imposed in Poland from March to April 2020, with a ban on access to forests and parks, reduced the risk of exposure to tick bites. In our opinion, given the course of symptomatic TBE [64,67], the limitation of access to primary and specialist healthcare may have had minor importance, although other authors emphasize this factor [32,68,69]. Our opinion is confirmed by the results of the analyses showing no correlations between the availability of medical personnel and TBE incidence, or between the COVID-19 and TBE incidence rates in the analyzed countries. Symptomatic patients infected with TBEV require medical care and, most often, hospitalization [26,70]. Due to the course of the disease, a “substantial delay” in the diagnosis and treatment of TBE should be ruled out. However, the incomplete reporting due to the large bureaucratic burden posed on the health service by the pandemic may have influenced the official number of TBE cases in government reports.

## 5. Conclusions

Based on the long-term period (2015–2019), Central Europe and the Baltic Sea countries are areas of high TBEV infection risk. Despite the COVID-19 pandemic in 2020 and the resulting restrictions, an increase in the TBE incidence rate was observed in more than half of the analyzed countries. In the pandemic year (2020), the highest increase in TBE incidence rate was observed in countries with different climatic zones, including France, Bulgaria, Norway, Austria and Italy. We highly recommend future research that focus on TBE incidence in the context of the COVID-19 pandemic.

## Figures and Tables

**Table 1:** Healthcare personnel, COVID-19 incidence in 2020, and TBE incidence in 2015–2020 in the analyzed countries.

Country	Health Care Personnel per 100,000	Incidence of COVID-19 per 100,000 in 2020	Number of Cases and Incidence of TBE per 100,000 Inhabitants											
			2015		2016		2017		2018		2019		2020	
			N	Inc.	N	Inc.	N	Inc.	N	Inc.	N	Inc.	N	Inc.
Austria	1612.14	4040.5	79	0.92	96	1.10	123	1.40	170	1.93	106	1.20	250	2.81
Belgium	1296.33	5587.7	1	0.01	1	0.01	3	0.03	3	0.03	4	0.03	7	0.06
Bulgaria	nd.	2922.9	2	0.03	0	0.00	1	0.01	0	0.00	1	0.01	2	0.03
Croatia	nd.	5218.7	26	0.62	6	0.14	10	0.24	22	0.54	13	0.32	14	0.34
Czech Republic	1400.43	6716.5	349	3.31	565	5.35	677	6.40	712	6.71	773	7.26	849	7.94
Estonia	994.67	2104.5	115	8.75	80	6.08	84	6.38	85	6.44	82	6.19	70	5.27
Finland	nd.	652.9	68	1.24	61	1.11	82	1.49	79	1.43	69	1.25	91	1.65
France	1754.85	3880.3	10	0.02	15	0.02	2	0.01 *	25	0.04	4	0.01	46	0.07
Germany	1262.44	2117.0	221	0.27	353	0.43	486	0.59	583	0.70	444	0.53	705	0.85
Greece	nd.	1300.1	1	0.01	0	0.00	0	0.00	2	0.02	0	0.00	0	0.00
Hungary	1002.61	3314.6	22	0.22	14	0.14	14	0.14	30	0.31	17	0.17	18	0.18
Ireland	1258.12	1831.9	0	0.00	0	0.00	0	0.00	0	0.00	1	0.02	0	0.00
Italy	nd.	3555.8	5	0.01	48	0.08	24	0.04	39	0.06	37	0.05	55	0.09
Latvia	nd.	2164.2	141	7.10	91	4.62	178	9.13	100	5.17	118	6.15	149	7.81
Lithuania	1628.41	5069.8	336	11.5	633	21.91	474	16.64	384	13.67	711	25.45	679	24.3
Luxembourg	nd.	7367.5	1	0.18	0	0.00	0	0.00	0	0.00	0	0.00	0	0.00
Norway	1906.19	919.6	9	0.17	12	0.23	16	0.30	26	0.49	35	0.66	41	0.76
Poland	nd.	3422.0	115	0.30	211	0.56	196	0.52	148	0.39	197	0.52	114	0.3
Romania	946.67	3294.8	0	0.00	0	0.00	1	0.01	4	0.02	0	0.00	0	0.00
Slovakia	793.44	3288.3	80	1.48	169	3.11	75	1.38	156	2.87	161	2.95	185	3.39
Slovenia	nd.	5789.2	62	3.01	83	4.02	102	4.94	153	7.40	111	5.33	187	8.92
Spain	nd.	4068.9	0	0.00	0	0.00	0	0.00	0	0.00	1	0.01 *	0	0.00
Sweden	nd.	4213.7	268	2.75	238	2.42	365	3.65	359	3.55	355	3.47	267	2.59

nd.—no data, N—number of reported cases of TBE, Inc.—incidence of TBE per 100,000 inhabitants, * incidence < 0.01.

**Table 2:** Comparisons and the level of the statistical significance of differences in the TBE incidence in 2015–2019 and in 2020 in the analyzed countries.

Country	Incidence of TBE per 100,000 Inhabitants		Statistical Analysis	
	2015–2019	2020	*t* (df = 4)	*p*
Austria	1.31	2.81	−8.65	0.0010
Belgium	0.02	0.06	−7.76	0.0015
Bulgaria	0.01	0.03	−3.65	0.0217
Croatia	0.37	0.34	0.35	0.7414
Czech Republic	5.81	7.94	−3.06	0.0376
Estonia	6.77	5.27	3.00	0.0400
Finland	1.30	1.65	−5.02	0.0074
France	0.02	0.07	−7.84	0.0014
Germany	0.50	0.85	−4.74	0.0091
Greece	0.01	0.00	1.50	0.2080
Hungary	0.20	0.18	0.50	0.6436
Ireland	0.00	0.00	1.00	0.3739
Italy	0.05	0.09	−3.63	0.0222
Latvia	6.43	7.81	−1.73	0.1590
Lithuania	17.83	24.30	−2.50	0.0666
Luxembourg	0.04	0.00	1.00	0.3739
Norway	0.37	0.76	−4.32	0.0124
Poland	0.46	0.30	3.24	0.0318
Romania	0.01	0.00	1.50	0.2080
Slovakia	2.36	3.39	−2.71	0.0537
Slovenia	4.94	8.92	−5.42	0.0056
Spain	0.00	0.00	-	-
Sweden	3.17	2.59	2.36	0.0780

*t*—result of the statistical test, *p*—probability, df—degrees of freedom.

## Data Availability

The raw data are available from the corresponding author.
